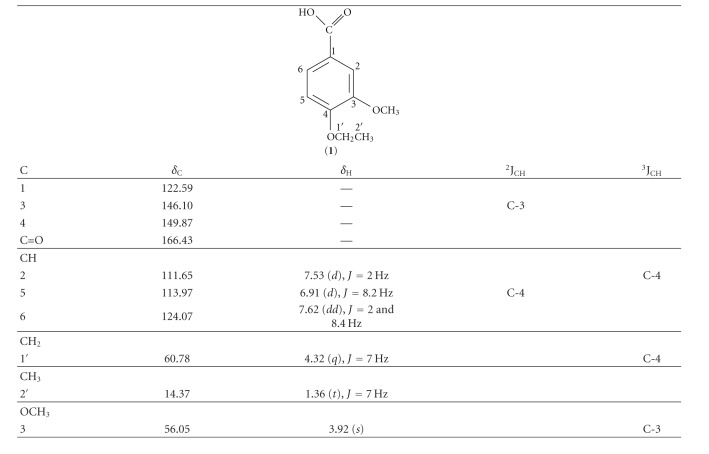# Antinociceptive Activity of Ethanol Extract from *Duguetia chrysocarpa* Maas (Annonaceae)

**DOI:** 10.1100/2012/859210

**Published:** 2012-05-03

**Authors:** Jackson Roberto Guedes da Silva Almeida, Edigênia Cavalcante da Cruz Araújo, Luciano Augusto de Araújo Ribeiro, Julianeli Tolentino de Lima, Xirley Pereira Nunes, Ana Sílvia Suassuna Carneiro Lúcio, Maria de Fátima Agra, José Maria Barbosa Filho

**Affiliations:** ^1^Núcleo de Estudos e Pesquisas de Plantas Medicinais, Universidade Federal do Vale do São Francisco, 56.304-205 Petrolina, PE, Brazil; ^2^Laboratório de Tecnologia Farmacêutica, Universidade Federal da Paraíba, 58.051-970 João Pessoa, PB, Brazil

## Abstract

The ethanol extract from the fruits of *Duguetia chrysocarpa* was evaluated for its antinociceptive activity in chemical and thermal models of nociception in mice. The intraperitoneal administration of the ethanol extract (100, 200, and 400 mg/kg body weight) showed a dose-dependent inhibition of acetic-acid-induced abdominal writhes. The extract also produced a significant inhibition of both phases of the formalin test in all doses tested and increased the reaction time in hot-plate test at dose of 200 mg/kg. The data obtained suggest that the antinociceptive effect of the extract may be mediated via both peripheral and central mechanisms. The phytochemical investigation yielded the isolation of the benzenoid derivative 3-methoxy-4-ethoxy benzoic acid which is being reported for the first time in this genus.

## 1. Introduction

Pain is a sensorial modality which in many cases represents the only symptom for the diagnosis of several diseases. It often has a protective function [1]. Pain is one of the most pervasive problems in our society and has high social costs due to the significant impairment or permanent disabling of millions of people. Pain can be defined as an unpleasant perception of a nociceptive sensation. This concept involves 2 components, nociception and perception. Pain perception is an integrative function modulated by emotional, motivational, psychological conditions and individual's past history. Nociception or the nociceptive sensation results from the activation of specific primary sensory neuron subpopulations that transmit the nociceptive information to the spinal cord from where it is relayed to supra spinal levels [2, 3].

In a general manner, there are four types of pain: (a) nociceptive pain, due to excessive stimulation of nociceptors localized in the skin, viscera, and other organs; (b) neurogenic pain, pain reflecting damage to neuronal tissue in the periphery or CNS; (c) neuropathic pain, due to a dysfunction of, or damage to, a nerve or group of nerves; (d) psychogenic pain, not due to an identifiable, somatic origin and which may reflect psychological factors. Pain is usually elicited by the activation of specific nociceptors (nociceptive pain). However, it may also result from injury to sensory fibres or from damage to the CNS itself (neuropathic pain) [4].

Despite the progress that has occurred in recent years in the development of pain therapy, there is still a need for effective and potent analgesics, especially for the treatment of chronic pain [5]. Plant-derived substances have, and will certainly continue to have, a relevant place in the process of extract discovery, particularly in the development of new analgesics [6].

The use of medicinal plants as analgesic and anti-inflammatory drugs in folk medicine is a practice common in many countries, although, in most cases, the active principles of the plants are unknown. However, evaluation of the pharmacological effects of the herbal crude extracts can still be used as a logical research strategy for searching of new drugs [7].

Annonaceae is a large family comprising *ca*. 135 genera and 2500 species which are distributed mainly in tropical and subtropical regions of the world [8]. Chemical studies with species of this family have reported the isolation of terpenoids (mainly diterpenes), essential oils which the composition is predominantly of monoterpenes and sesquiterpenes, and alkaloids, especially isoquinoline alkaloids [9].

The genus *Duguetia* consists of approximately 80 known species native to tropical America. Few chemical data are available on this genus, despite the considerable number of species. Several isoquinoline-derived alkaloids and sesquiterpene-type structures have been reported [10, 11]. Chemical study realized with species of this genus by our group showed the isolation of alkaloids and a new cinnamate derivative from *Duguetia gardneriana *[12]. The chemical composition and antimicrobial activity of the leaf essential oils of *Duguetia gardneriana* and *Duguetia moricandiana* also were evaluated [13]. Discretamine, an alkaloid isolated from *Duguetia moricandiana,* demonstrated antinociceptive activity in experimental models [14]. In a recent study, we evaluated the phenolic quantification and antioxidant activity of *Anaxagorea dolichocarpa* and *Duguetia chrysocarpa *[15].

There is no previous report on pharmacological studies of *D. chrysocarpa*. Thus, the aim of this study was to evaluate the antinociceptive effect of ethanol extract from *D. chrysocarpa* in experimental models of nociception.

## 2. Materials and Methods

### 2.1. Plant Material

The fruits of *Duguetia chrysocarpa* Maas were collected in Santa Rita, State of Paraiba, Brazil, in January 2004. A voucher specimen (AGRA 5538) was deposited at the Herbarium Professor Lauro Pires Xavier (JPB), of the Federal University of Paraiba.

### 2.2. Preparation of Plant Extract

The fruits of *D. chrysocarpa* (2000 g), dried and pulverized, were subjected to maceration with 95% EtOH for 72 hours. The EtOH solution was concentrated under vacuum yielding 107 g of crude ethanol extract of *D. chrysocarpa* (Dc-EtOH).

### 2.3. Preliminary Phytochemical Screening

Preliminary phytochemical analysis of the extract was carried. The presence of alkaloids was tested with Dragendorff's and Mayer's reagents, flavonoids with HCl and Mg powder, phenols with ferric chloride, and steroids and terpenoids by Liebermann-Burchard reaction [16].

### 2.4. Animals

Male adult albino Swiss mice (35–40 g) were used throughout this study. The animals were randomly housed in appropriate cages at 22 ± 2°C on a 12 h light/dark cycle with free access to food and water. When necessary, animals were deprived of food 12 h prior to the experiments. They were used in groups of six animals each. All nociception tests were carried out by the same visual observer. Experimental protocols and procedures were approved by the Universidade Federal do Vale do São Francisco Animal Care and Use Committee by number 024240408.

### 2.5. Pharmacological Tests

#### 2.5.1. Acetic-Acid-Induced Writhing Test

The test was performed as described by Koster et al. [17] with modifications. Mice were divided into six groups of six mice each and pretreated with vehicle (saline), morphine (10 mg/kg), acetylsalicylic acid (ASA 150 mg/kg), and Dc-EtOH (100, 200, and 400 mg/kg) 30 min before acetic acid injection (0.9% v/v) i.p. in a volume of 0.1 mL/10 g. The number of abdominal constrictions (full extension of both hind paws) produced in each group for the succeeding 10 min was counted and compared to the response in the control group. The antinociceptive activity was expressed as percentage of inhibition of the abdominal constrictions.

#### 2.5.2. Formalin Test

The formalin test was carried out as described by Hunskaar and Hole [18]. Vehicle (saline), morphine (10 mg/kg), acetylsalicylic acid (ASA 150 mg/kg) and Dc-EtOH (100, 200, and 400 mg/kg) were administered i.p. 60 min before formalin injection. 20 *μ*L of 2.5% formalin solution (0.92% formaldehyde) in 0.9% saline were injected subcutaneously into the right hind paw of mice. Mice were observed in the chambers with a mirror mounted on three sides to allow view of the paws and the amount of time (in seconds) that the animal spent licking the injected paw was considered as an indicative of pain. Two distinct phases of intensive licking activity were identified. Responses were measured for 5 min after formalin injection (first phase, neurogenic) and 15–30 min after formalin injection (second phase, inflammatory).

#### 2.5.3. Hot Plate Test

In the hot-plate test, mice were preselected on the hot plate at 55 ± 0.5°C. Licks on the rear paws were the parameters of observation. Animals showing a reaction time (latency for licking the hind feet or jumping) greater than 20 s were discarded. The animals were then treated with vehicle (saline, 0.1 mL/10 g), morphine (10 mg/kg), and Dc-EtOH (100, 200, and 400 mg/kg) via i.p. The reaction time (in seconds) for each mice was determined on the hot plate during the maximum period of 20 s, at intervals of 30, 60, 90, and 120 min after the administration of the extract [19].

### 2.6. Statistical Analysis

All data were expressed as mean ± SEM. and the statistical significance was determined using an analysis of variance (ANOVA) followed by Dunnett's multiple comparison test. Values were considered significantly different at *P* < 0.05. All analysis was performed using GraphPad Prism 4.0 program.

### 2.7. Isolation of a Compound from *D. chrysocarpa*

The ethanol extract was acidified with 3% HCl and subjected to extraction of alkaloids, obtaining a chloroform phase and the fraction of total tertiary alkaloids. The chloroform phase (3 g) was chromatographed on silica gel column in which 255 fractions were obtained. These fractions were monitored by TLC. The fraction 5-6, after analysis by TLC, was submitted for spectral analysis of ^1^H and ^13^C NMR using one- and two-dimensional methods. ^1^H and ^13^C-NMR spectra were measured at 200 MHz for ^1^H and 50 MHz for ^13^C, using CDCl_3_ as solvent and TMS as an internal standard.

## 3. Results

### 3.1. Preliminary Phytochemical Screening

Preliminary analysis demonstrated that Dc-EtOH was found to be positive for the presence of alkaloids, steroids, and terpenoids. However, the ethanol extract was found to be negative for the presence of flavonoids.

### 3.2. Acetic-Acid-Induced Writhing Test

The intraperitoneal administration of Dc-EtOH (100, 200, and 400 mg/kg) had a significant effect (*P* < 0.01) on the number of writhing induced by the i.p. administration of the acetic acid as compared with control group treated with saline (Table 1). The effect was more significant for morphine, which abolished the abdominal writhings.

### 3.3. Formalin Test

Table 2 shows the results obtained with the formalin test. During the first phase (0–5 min) and second phase (15–30 min), Dc-EtOH showed a significant reduction the licking activity in all doses tested.

### 3.4. Hot-Plate Test

In the hot-plate test, the animal treated with ethanol extract of *D. chrysocarpa* at dose of 200 mg/kg modified the latency time 90 and 120 min after the administration of extract when compared with control animals (*P* < 0.05). The effect of morphine (10 mg/kg) was significantly higher (Table 3).

### 3.5. Structure Determination of a Compound Isolated from *D. chrysocarpa*

The analysis of all the spectral data for the compound and comparison with values of literature led to the determination of its structure as 3-methoxy-4-ethoxy benzoic acid **(1)**. This compound was isolated previously from *Angelica furcijuga *[20] but described here for the first time in the genus *Duguetia*. The 1D and 2D NMR spectra were also used to assign unambiguously the ^1^H and ^13^C chemical shifts (Table 4).

## 4. Discussion 

Pain is a sensorial modality, which in many cases represents the only symptom for the diagnosis of several diseases, and often has a protective function. Throughout history, man has used many different forms of therapy for the relief of pain, and medicinal herbs are highlighted due to their popular use [21]. 

In this study, we aimed to investigate the possible antinociceptive effect of fruits ethanol extract from *Duguetia chrysocarpa* (Dc-EtOH) using chemical and thermal models of nociception. The first test to evaluate the antinociceptive activity of Dc-EtOH was the writhing induced by acetic-acid, which is used to screen for both peripherally and centrally acting agents [19]. Dc-EtOH significantly reduced in a dose-dependent manner the acetic acid-induced writhing in mice. Intraperitoneal injection of acetic acid produced 17.80 ± 2.29 writhes in the control group for 10 min after injection. The groups previously treated with 100, 200, and 400 mg/kg of Dc-EtOH exhibited a significant reduction in the number of writhings of 24.72, 62.92, and 71.92%, respectively. The results revealed that Dc-EtOH has a potent antinociceptive activity in this method. Collier et al. [22] postulated that the acetic acid acts indirectly by inducing the release of endogenous mediators which stimulate the nociceptive neurons sensitive to nonsteroidal anti-inflammatory drugs (NSAIDs) and opioids. One possible mechanism of antinociceptive activity of Dc-EtOH could be due to the blockade of the effect or the release of endogenous substances (arachidonic acid metabolites) that sensitize and activate peripheral nociceptors. The result of this test, however, does not ascertain whether the antinociceptive effect was mediated by central or peripheral process [23]. 

In order to distinguish between the central and peripheral antinociceptive action, the formalin test was performed. Formalin is a noxious stimulus commonly used in animal behavioral experiments. The formalin test originally described by Dubuisson and Dennis [24] (1977) consists of a subcutaneous (s.c.) formalin injection into the rat hind paw that produces a biphasic nociceptive response which is responsive to many classes of analgesic drugs [18]. The typical time course of the behavioral and electrophysiological responses to formalin consists of an early phase of short-lasting response, followed by a continuous prolonged response that mimics some features of postinjury pain in man [25]. The initial response (phase 1) is initially attributed to a direct algogenic effect of formalin on the nociceptors, whereas phase 2 is associated with the release of local endogenous mediators responsible for sensitization of primary and spinal sensory neurons and subsequent activation of the nociceptors [26]. In this experiment, Dc-EtOH decreased the licking time in mice in both phases, suggesting that this extract exerts its antinociceptive effect by central mechanisms. The extract at dose of 400 mg/kg was more effective in the second phase, with percentage of inhibition of 89.12%, suggesting at least in part a possible peripheral mechanism. 

The effect of Dc-EtOH on hot-plate response provides a confirmation of its central effect. In this model, it also significantly increased the latency time at dose of 200 mg/kg. As the hot-plate test is a specific central antinociceptive test, it is possible that the extract exert their effects through central mechanisms which are then investigated. 

In a previous study [27], the essential oil from barks of *Duguetia lanceolata* showed antinociceptive and anti-inflammatory effects and probably the mechanisms(s) involve central and peripheric actions. Thus, the genus *Duguetia* presents a great potential for the discovery of new molecules with antinociceptive activity. 

## 5. Conclusions

In conclusion, the results show that *Duguetia chrysocarpa* possesses a good antinociceptive potential. The presence of alkaloids may be related to the effect of this extract as many alkaloids show properties on the central nervous system. The isolation of a derivative of benzoic acid also contributes to the chemotaxonomy of the genus *Duguetia*. Further studies will be performed for the isolation of new chemical constituents of the plant as well as for a better understanding of the mechanism of antinociceptive activity presented by the extract. 

## Figures and Tables

**Table 1:** Antinociceptive effect of Dc-EtOH on acetic acid-induced writhing in mice.

Group	Dose (mg/kg)	No. of writhings	% inhibition
Control	—	17.80 ± 2.29	—
Dc-EtOH	100	13.40 ± 2.50**	24.72
	200	6.60 ± 2.54**	62.92
	400	5.00 ± 1.61**	71.92
Morphine	10	0.0 ± 0.0**	100
Acetylsalicylic acid	150	2.17 ± 0.87**	87.80

Values are mean ± SEM, *n* = 6. **P < 0.01 significantly different from control (ANOVA followed by Dunnett's test).

**Table 2:** Effect of Dc-EtOH on formalin-induced pain in mice.

Group	Dose (mg/kg)	First phase		Second phase	
		Licking time (s)	% inhibition	Licking time (s)	% inhibition
Control	—	72.40 ± 5.77	—	81.17 ± 4.32	—
Dc-EtOH	100	32.00 ± 5.05**	55.80	57.83 ± 5.82**	28.75
	200	44.00 ± 1.79**	39.22	62.80 ± 4.12**	22.63
	400	37.20 ± 4.95**	48.62	8.83 ± 3.03**	89.12
Morphine	10	9.80 ± 4.72**	86.46	30.00 ± 2.94**	63.04
Acetylsalicylic acid	150	22.40 ± 3.11**	69.06	13.17 ± 10.25**	83.77

Values are mean ± SEM, *n* = 6. **P < 0.01 significantly different from control (ANOVA followed by Dunnett's test).

**Table 3:** Effect of Dc-EtOH on hot-plate test in mice.

Group	Dose (mg/kg)	Latency time (s)			
		30 min	60 min	90 min	120 min
Control	—	5.10 ± 1.10	7.90 ± 1.50	5.30 ± 0.20	5.40 ± 0.60
Dc-EtOH	100	5.20 ± 1.34	6.20 ± 1.02	5.40 ± 0.87	5.80 ± 1.02
	200	5.00 ± 0.80	8.00 ± 1.20	8.00 ± 1.10*	9.40 ± 1.40*
	400	4.40 ± 0.40	5.70 ± 1.10	5.40 ± 0.80	6.10 ± 0.80
Morphine	10	19.33 ± 0.67**	18.00 ± 1.23**	17.80 ± 1.11**	14.80 ± 0.86**

Values are mean ± SEM, *n* = 6. *P < 0,05; **P < 0.01 significantly different from control (ANOVA followed by Dunnett's test).

**Table 4:** ^1^H (200 MHz) and ^13^C (50 MHz) NMR data for 1 including results obtained by heteronuclear 2D shift-correlated HMQC and HMBC spectra, in CDCl_3_ as solvent and TMS as internal reference. Chemical shifts in *δ* (ppm) and coupling constants (J, in parenthesis) in Hz.